# Comparison of Ethanol Stability and Chemical Composition of Camel Milk from Five Samples

**DOI:** 10.3390/ani12050615

**Published:** 2022-03-01

**Authors:** Omar A. Alhaj, Roua Lajnaf, Zeineb Jrad, Mohammad A. Alshuniaber, Haitham A. Jahrami, Mohamed F. Serag El-Din

**Affiliations:** 1 Department of Nutrition, Faculty of Pharmacy and Medical Sciences, University of Petra, Amman 11196, Jordan; omar.alhaj@uop.edu.jo; 2 Department of Biological Engineering, National Engineering School of Sfax, Sfax 3038, Tunisia; roua_lajnaf@yahoo.fr; 3 Unit of Agropolymers Engineering and Emerging Technologies UMR IATE, University of Montpellier, CEDEX 5, 34095 Montpellier, France; 4 Livestock and Wildlife Laboratory, Arid Regions Institute (IRA) Médenine, University of Gabes, Gabes 4100, Tunisia; jradzeineb@yahoo.fr; 5 Department of Food Sciences and Nutrition, College of Food and Agriculture Sciences, King Saud University, Riyadh 11451, Saudi Arabia; 6 Ministry of Health, Manama 410, Bahrain; hjahrami@health.gov.bh; 7 College of Medicine and Medical Sciences, Arabian Gulf University, Manama 323, Bahrain; 8 Department of Nutrition and Food Science, Faculty of Home Economics, Menofia University, Shibin El Kom 11925, Egypt; serag_m1956@yahoo.com

**Keywords:** camel milk, milk proteins, ethanol stability, chemical composition

## Abstract

**Simple Summary:**

Camel milk has recently gained the interest of consumers and the dairy industry, as it is widely suggested as an ideal substitute for cow milk. The nutritional value and the bioactivity of camel milk proteins have received particular attention from research groups and industrial companies around the world. Camel milk proteins can be used as ingredients in the manufacturing and stabilization of foods and beverages; however, in these applications, the controlled aggregation of milk proteins and stability at high temperatures and in alcohol are desirable. The ethanol stability of milk could be used as an indicator of its freshness and provide information on the stability of raw milk ultra-high temperatures and powder processing.

**Abstract:**

This research was carried out to study the variation in ethanol stability and chemical composition of five camel milk samples, including two pasteurized samples (Alwatania and Darir alabaker) and three raw samples (Majaheim, Wadah, and Hamra). Ethanol stability was analyzed by dispersing camel milk samples with 0 to 100% ethanol (*v*/*v*). The findings indicate that camel milk samples precipitated after adding an equal volume of ethanol at concentrations between 50% and 64% ethanol, depending on the milk sample. The addition of sodium chloride at different concentrations (1–10%) to camel milk resulted in a significant increase in ethanol stability, and samples from Majaheim and Alwatania exhibited the highest ethanol stability values (88%). In contrast, the addition of EDTA to camel milk for pH ranging between 5.9 and 7.1 has increased ethanol stability with a sigmoidal shape in camel milk. The largest ethanol stability differences were observed in a camel milk sample from Alwatania. Thus, the level of Ca^2+^ in camel milk may contribute to ethanol stability by shifting the entire profile to higher ethanol stability values. The chemical composition of different camel samples was also determined. The lactose content of camel milk varied significantly (*p* < 0.05) across samples, ranging from 4.37% in Majaheim camel milk to 4.87% in Alwatania camel milk. The total solids of camel milk varied significantly between raw and pasteurized samples, ranging between 10.17% and 12.10%. Furthermore, protein concentration in camel milk obtained from different camel samples varied, from 2.43% to 3.23% for Hamra and Alwatania, respectively. In conclusion, ethanol stability in camel milk was dependent on the camel breed, pH level, ionic strength, and EDTA addition.

## 1. Introduction

Mother’s milk of different species is a high-quality source of nutrients at the early stages of life. Cow milk is the most commonly consumed and processed milk. Consumption of milk from other animals, such as camel milk, is becoming more widespread. Camel milk has recently gained the interest of consumers and the dairy industry, as it is widely suggested as an ideal substitute for cow milk [1]. Recently, the presence of alcohol (ethanol) and/or heat during the dispersion of sodium caseinate was found to have a significant effect on protein solubility but have no effect on the physical parameters of protein’s aggregations, such as size and surface charge [2].

In camel milk, despite the low ratio of casein to whey proteins, the casein fraction is mainly a substrate for bioactive peptide generation [3,4,5]. Camel milk caseins are primarily composed of β-casein, followed by α-casein and κ-casein (65%, 21%, and 3.47% of total casein, respectively) [6]. The camel milk whey proteins are predominantly α-lactalbumin, lactoferrin, camel serum albumin, peptidoglycan recognition protein, and immunoglobulins [7]. In contrast to bovine milk, β-lactoglobulin, which is a common allergen, is deficient in camel’s milk [8,9]. This deficiency, combined with the abundance of β-casein, ensures that camel milk is easier to digest and less allergenic than cow’s milk, making it tolerable for people suffering from allergic symptoms [10]. 

The nutritional value and the bioactivity of camel milk proteins have received particular attention from the scientific community and industrial companies around the world. Camel milk or proteins can be used as ingredients in the manufacturing and stabilization of foods and beverages; however, in these applications, controlled aggregation of milk proteins and stability at high temperatures and in alcohol is desirable. In the past, the ethanol stability of milk was used as an indicator of its freshness and to provide information on the stability of raw milk at ultra-high temperatures and powder processing [11,12]. Thus, alcohol testing was extremely useful to the global dairy industry since it allowed acidic milk, such as colostrum or mastic milk, to be processed without causing quality issues or coagulation in the dairy pasteurizer’s heating plates [13]. Much attention has been devoted to the heat stability of camel milk in recent years [14], but there is relatively little published information on the effects of ethanol on camel milk protein stability. 

Ethanol stability is defined as the minimum concentration of added aqueous ethanol that results in milk coagulation. It is related to the chemical properties of milk, including pH, divalent cation content, and saline balance. The ethanol stability of cow milk is pH dependent with a typical sigmoidal pH profile; increasing the pH value of milk increases its ethanol stability.

Regarding casein fraction, the addition of ethanol to milk allows for interaction between charges on the κ-casein layer by reducing the dielectric constant of the medium [15], which decreases the negative micellar charges and their repulsion force and then promotes milk coagulation. Hence, it is relevant to know how milk proteins are destabilized by ethanol from a technological point of view. Furthermore, Horne [12] and Rosa et al. [13] reported that ethanol stability differs from one species to another. In comparison to cow milk, there is a scarcity of current understanding in the alcohol stability of camel milk. Sagar et al. [16] reported that camel milk has negative alcohol stability, though no visible flakes nor coagulation formation were reported. On the other hand, in other studies, the addition of salts and increasing pH values were found to improve the alcohol stability of camel milk to up to 85% [16,17]. Furthermore, the addition of EDTA to camel milk can improve ethanol stability [18]; moreover, it could convert sterilized camel milk from type A to B [19]. Therefore, we can correlate the particular ethanol stability of camel’s milk based on its chemical and protein profiles. The assessment of camel milk quality before processing is becoming increasingly important due to the recent increase in interest from consumers and the dairy industry. In this respect, this study aims to compare the chemical composition of camel milk collected from five samples and furthermore provide additional evidence on the effect of adding NaCl at different pH values and EDTA (ethylene diamine tetra acetic) levels by removing Ca^2+^ on the ethanol stability.

## 2. Materials and Methods

### 2.1. Milk Sample Collection

A total of 12–15 pasteurized camel milk samples and 28–35 raw camel milk samples (originated randomly from 7 to 10 lactating females) of different breeds were collected and then tested between April and June of 2021. One-liter samples were included from two commercially available brands of pasteurized (at 75 °C for 15 s) whole camel milk (Alwatania and Darir alabaker) purchased from the local market in the city of Riyadh (in the central region of Saudi Arabia). In addition, 2 L of raw samples was collected from a local private farm in the central region of Saudi Arabia. These samples included milk from three major breeds of camels (*Camelus dromedarius*) (Majaheim, Wadah, and Hamra) for comparison. Fresh morning milk samples were collected from healthy animals (individual camels) and during the same stage of lactation (from 10 to 16 weeks). All camels were fed approximately the same diet on the farm, including fresh-cut grass (alfalfa), hay, and mixed grain concentrate. After collection, fresh milk samples were immediately transported to the laboratory in an icebox at 4 °C for analysis.

### 2.2. Chemical Composition

#### 2.2.1. Protein Determination

The standard Kjeldahl method of the AOAC [20] was used to determine the nitrogen concentration in milk. In the Kjeldahl procedure, after digestion in concentrated sulfuric acid (15 mL), the total organic nitrogen in a volume of 5 mL of milk is converted to ammonium sulfate. Under alkaline conditions, ammonia is formed and distilled into boric acid solution. The formed borate anions are titrated with standardized hydrochloric acid, and the nitrogen content representing the amount of crude protein in the studied milk sample is calculated. Protein was finally calculated by multiplying the nitrogen concentration by a conversion factor of 6.38.

#### 2.2.2. Mineral Determination

Calcium and sodium concentrations were determined by dissolving in 2% HCl, then analyzed using a flame atomic absorption spectrophotometer (PerkinElmer Model 3110, Norwalk, CT, USA). Lanthanum (1%) was added to the final calcium dilution to overcome phosphate interference with calcium. A full set of standards (Sigma-Aldrich, St. Louis, MO, USA) was analyzed prior to analysis of the samples.

#### 2.2.3. Fat Determination

The Gerber acid butyrometer method was used to determine fat concentrations in the milk samples. Milk fat is separated from proteins by adding sulfuric acid (10 mL), and the separation is facilitated using amyl alcohol (1 mL) and centrifugation for 3–5 min. The fat content is read directly via a special calibrated butyrometer. The milk volume used in each measurement is about 11 mL, and the results were expressed as percentages (%, *w*/*v*).

#### 2.2.4. Total Solids Content

Total solids concentrations, expressed in g/L of milk, were determined by weighing the samples dried at 105 °C for 6 h. A volume of 4 mL of camel milk samples for each test was used to determine the total solid content [21].

#### 2.2.5. Lactose Determination

The content of lactose was determined using the method described by Abu-Lehia [22]. A total of 5 mL of fluid milk was mixed thoroughly with 5 mL of TCA. Then, the mixture was filtered through 40 filter paper, while 2 mL of the clear filtrate was diluted to 100 mL with distilled water. A 1.0 mL aliquot of the diluted filtrate was transferred to a 15 mL screw-cap tube. To the contents of the tube, a 2.5 mL aliquot of the diluted working solution was added and thoroughly mixed. The tube was stoppered and placed in a vigorously boiling water bath for exactly 2.5 min, to a depth of 4 to 6 cm. The tube was then quickly cooled under cold tap water before being filled with 7 cc of distilled water and well mixed. The absorbance at 520 nm was compared to a blank that contained all of the reagents except for the milk, which was replaced with distilled water.

### 2.3. Determination of the Ethanol Stability of Milk

The ethanol stability of the milk samples was measured using the method described by Guo et al. [23]. Using this method, 5 mL of milk sample was mixed in a glass petri dish with an equal volume (5 mL) of ethanol solution ranging from 10% to 100% (*v*/*v*) at room temperature, and all samples in the current study were run in triplicate. The effects of adding sodium chloride (NaCl) at different concentrations and pH ranging between 5.9 and 7.1 on ethanol stability at room temperature were measured. The concentration of sodium chloride was ranging between 1 and 10%. EDTA was added to milk samples at a concentration of 6.0 mmol/L before the ethanol stability test, and 1 milliliter of EDTA solution can chelate 0.1 milligram of Ca^2+^ [18]. The pH of milk samples was adjusted by adding appropriate amounts of 1 M HCl or NaOH. The maximum concentration of aqueous ethanol that failed to cause the milk to coagulate was defined as the ethanol stability point. This study was designed according to Guo et al. [23] and Zhao et al. [18].

### 2.4. Statistical Analysis

The data were analyzed using SPSS software for Windows, version 10.0 (IBM, Armonk, NY, USA), and are reported as means and standard deviations (SDs). The ANOVA test was used to analyze variation between groups, followed by Duncan’s multiple range test. The level of statistical significance was set at *p* ≤ 0.05.

## 3. Results

### 3.1. Chemical Composition

The chemical compositions of camel milk samples are presented in Table 1. The concentrations of fat, protein, lactose, total solids, and minerals varied significantly (*p* < 0.05) among camel milk samples. Fat concentration in milk ranged from 1.83% to 3.07% in camel milk samples.

No significant differences were observed in the fat concentrations of Alquasim and Alwatania camel milk samples. However, the fat concentrations in Majaheim, Wadah, and Hamra camel milk samples were significantly lower than those in Alwatania and Alquasim camel milk, and Majaheim camel milk had the lowest values (1.83%). On the other hand, protein concentration in camel milk obtained from different camel samples varied, from 2.43% to 3.23% for Hamra and Alwatania, respectively (Table 1). Alwatania was significantly different from all other protein samples. While no significant difference was found between Alquasim and Majaheim as well as between Wadah and Hamra. As shown in Table 1, the lactose content of camel milk varied significantly (*p* < 0.05) across samples, ranging from 4.37% in Majaheim camel milk to 4.87% in Alwatania camel milk. The total mineral concentration (ash) of camel milk samples ranged from 0.78 to 0.80%. Significant differences in ash values were observed between different camel samples, where the Hamra sample has a greater value than the other samples (Table 1).

Table 1 shows that the values varied significantly between raw and heat-treated samples for the total solids in camel milk, ranging between 10.17% and 12.10%. Likewise, total solids of camel milk were significantly different in Alwatania and Alquasim (Darir alabaker) than other samples.

### 3.2. Calcium and Sodium Content

The sodium concentration (Table 1) in Wadah (261.18 ± 5.16 ppm) and Hamra (188.64 ± 2.19 ppm) camel milk samples were significantly different from the sodium concentration of other camel milk samples. On the other hand, the calcium content of Wadah and Hamra camel milk was higher than that of the other camel milk samples. Moreover, significant differences were found in calcium content between heat-treated and raw samples.

### 3.3. Ethanol Stability of Camel Milk Samples

#### 3.3.1. Effect of NaCl

**Figure 1:**
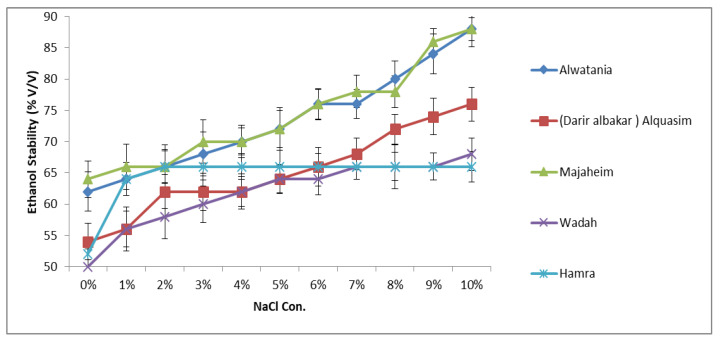
Effect of sodium chloride concentration (%: g/100 g) on ethanol stability of camel milk samples—Alwatania, Alquasim (Darir alabakar), Majaheim, Wadah, and Hamra (ethanol stability: % (*v*/*v*). Error bars indicate the standard deviation.

The ethanol stability results of the samples as a function of NaCl concentration are presented in Figure 1. The influence of ionic strength of NaCl on the ethanol stability of camel samples was studied by conducting experiments with solutions containing various amounts of NaCl (1–10%). As shown in Figure 1, the effect of NaCl on ethanol stability varied among camel samples. The addition of 1% (*w*/*v*) NaCl has increased the ethanol stability of camel milk from approximately 56% to 66% as shown in Figure 1. Analogously, as the concentration of NaCl increased up to 10% (*w*/*v*), the stability further improved. A similar profile was observed in Majaheim and Alwatania milk, which achieved the highest ethanol stability values (approximately 88%) of all camel milk samples regardless of sodium chloride concentration. The ethanol stability of Alquasim milk containing 7–10% (*w*/*v*) NaCl was higher than that of both Wadah and Hamra milk with the same added NaCl content. However, with the addition of NaCl between 1% and 5%, the ethanol stability was higher in Hamra camel milk (Figure 1).

#### 3.3.2. Effect of pH and Ca^2+^

**Figure 2:**
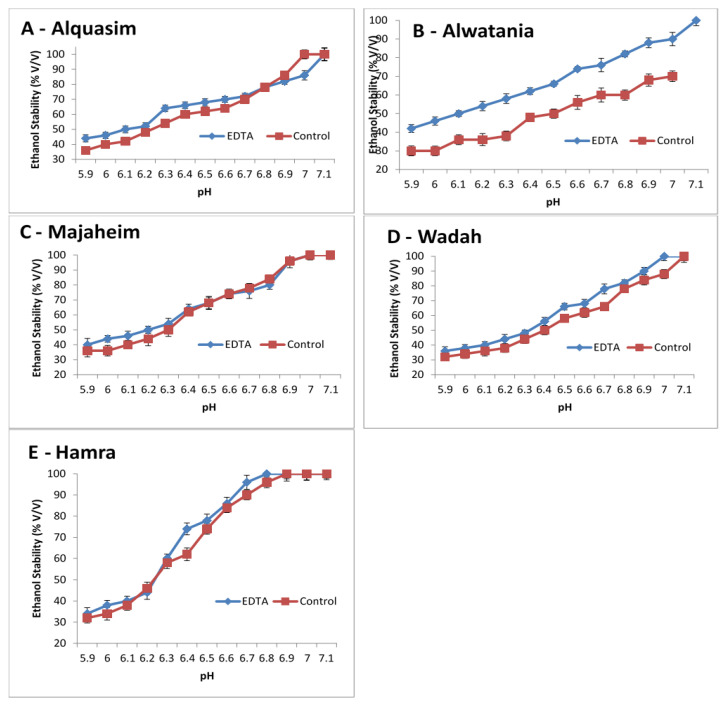
Changes in pH and ethanol stability of camel milk samples, Alquasim (**A**), Alwatania (**B**), Majaheim (**C**), Wadah (**D**), and Hamra (**E**) (ethanol stability: % (*v*/*v*)). Error bars indicate the standard deviation.

The effects of pH and the removal of calcium (using EDTA) were studied in camel milk as shown in Figure 2. The ethanol stability of camel milk increased when the pH was increased from 5.9 to 7.1, and it was higher for EDTA-treated milk than control milk (Figure 2). For camel milk from the Majaheim and Hamra breeds (Figure 2C,E), the stability was similar between the control and EDTA-treated milk, regardless of the pH value, except at a pH of 6.4, 6.7, and 6.9 for Hamra milk, where EDTA-treated milk presented higher ethanol stability than the control (74%, 96%, and 100%, respectively). Contrastingly, EDTA-treated milk in the Wadah milk sample (Figure 2D) exhibited greater ethanol stability values than control milk. For Alquasim milk (Darir alabakar), the ethanol stability values of EDTA-treated milk (Figure 2A) were higher when compared to control milk. However, at a pH level above 6.8, the control had the highest ethanol stability values (78%) compared to EDTA-treated milk. The stability of Alwatania camel milk (Figure 2B) increased when the pH increased and was considerably higher for EDTA-treated milk than control milk. The Alwatania camel milk sample demonstrated larger ethanol stability differences compared to milk from other heat-treated camel samples (Figure 2A), suggesting that Alwatania milk is more sensitive to EDTA treatment.

## 4. Discussion

### 4.1. Chemical Composition

The composition of camel milk has been studied in different parts of the world. Literature data show a wide range of camel milk composition, which is consistent with our findings. The obtained results of the fat content are consistent with those reported by previous authors El-Agamy [24], Haddadin et al. [25], and Shuiep et al. [26], which have estimated the fat content in camel milk at approximately 2.95%, lower than those reported by He et al. [27] (5.1–6.24%) for Bactrian camel milk and by Lajnaf et al. [28] (3.54%) for dromedary camel milk. Faye et al. [29] reported a significantly higher content of fat in *Camelus bactrianus* compared to *Camelus dromedarius* breeds. Mehaia et al. [30] found that Majaheim camel milk exhibited the highest fat content (3.22%) and Wadah camel milk had the lowest values (2.46%).

Camel breed was found to play a significant role in the protein content of camel milk. Camel milk from the same breed, such as Majaheim [31], had similar protein contents (both casein and whey proteins). Our results referring to protein content are comparable to those found by the previous researchers Ho et al. [32], Farah and Ruegg [33], Wangoh et al. [34], and Kamal et al. [35] but lower than the values reported by El-Agamy [25] and Mukasa-Mugerwa [36]. These authors reported that the protein content is estimated within 3.7 and 4.9%. Furthermore, Alwatania was found to possess the highest protein concentration of the collected camel milk samples. This is consistent with the findings of Konuspayeva [28] and Mehaia et al. [30], who reported that Majaheim camel milk had a higher protein concentration than milk from other camel breeds, such as Wadah and Hamra. On the other hand, Lajnaf et al. [28] noted that camel milk exhibited lower protein amounts when compared to that of bovine milk, with values of 2.2 and 2.8% for camel and cow milk, respectively. It has been reported [23] that ethanol stability is greatly dependent on the casein content in goat milk. Moreover, De Oliveira et al. [37] reported that the lower the protein content (casein) in milk, the lower the ethanol stability.

For the lactose concentrations, our findings are comparable to those reported by Lajnaf et al. [28], Konuspayeva [38], Sawaya et al. [31], Mehaia [30], and Guliye et al. [31], but higher than the values reported by Shuiep et al. [26], and lower than that of He et al. [27] for Bactrian camel milk. For instance, the findings of Shuiep et al. [26] indicated that the lactose content in dromedary camel milk is around 3.12%, while Sawaya et al. [31] reported that camel milk contains 4.4% lactose. As explained in previous studies, such variation in lactose concentration in camel milk is a result of the types of plants ingested by camels in the deserts [32]. Furthermore, lactose concentration in camel milk varies slightly for some dromedary camel breeds in different world regions [25,30,31]. Total solids values are in agreement with the results of Bittante et al. [39], Zou et al. [40], Farah and Ruegg [33], Mehaia et al. [41], and Farag et al. [42] but lower than those of Indra [43]; moreover, they are higher than those of He et al. [27] for Bactrian camel milk.

Previous studies found that the mineral content of camel milk samples varied significantly among breeds, including Majaheim, Wadah, and Hamra [30,31,44]. For instance, results showed that the sodium concentration in Wadah camel milk was substantially higher than in other camel milk samples. These observations are in agreement with those of Mehaia et al. [30], who reported that Wadah camel milk had the highest concentrations of Na 73.4 ± 4.5 mg/100 g. Meanwhile, Mehaia et al. [30] found that Majaheim camel milk had higher calcium and magnesium concentrations than Wadah and Hamra camel milk, with a calcium concentration of 120 ± 5.1, 109 ± 4.5 and 119 ± 6.7 mg per 100 g for Majaheim, Wadah, and Hamra camel milk, respectively.

According to Haddadin et al. [25] and Mehaia et al. [30], the variations in mineral content in camel milk can be attributed to various factors, including breed differences, feeding, water intake, and analytical procedures. Similarly, previous studies have reported that the mineral content of camel milk varies between breeds, including Majaheim, Wadah, Najdi, and Hamra camels [30,31].

### 4.2. Ethanol Stability of Camel Milk

In the current study, the ethanol stability of camel milk samples was varied in the presence of heat (pasteurization), NaCl, EDTA addition and ethanol. The ethanol stability of milk is a parameter that associated with other milk properties, such as acidity, citrate and phosphate concentrations, and ionic strength [45]. Variation in ethanol stability amongst the five camel milk samples regardless of NaCl concentration could be due to the differences in milk chemical compositions, camel breed, feeding, as well as heat stress [46]. The most stable sample to ethanol (Alwatania) presents the highest value of non-fat solids, whereas Na concentrations were higher in unstable samples (Hamra). Those observations agreed with those of Chavez et al. [47]. The pasteurization process has no effect on ethanol stability, but the variation of mineral level such as Na, which influences the ionic strength, was more important to alcohol stability. In fact, the addition of NaCl to camel milk increases its sodium and potassium balance, thereby increasing the stabilization of casein micelles. Guo et al. [23] reported that a low Na/K ratio is one of the factors contributing to poor ethanol stability in goat’s milk. Therefore, adding NaCl may increase ionic strength by increasing the hydration of casein micelles and improving its ethanol stability. Moreover, Malmgren [48] reported that the addition of salts and increasing pH values have improved the alcohol stability of camel milk to up to 85%.

Moreover, the increase in ethanol stability can be attributed to the particularity of camel casein micelle composition. In addition, casein micelles are also associated with colloidal particles with a stabilizing hairy brush of Ca-sensitive κ-casein on their surface. Therefore, added NaCl behaved essentially as Ca-chelating salt and competes with the casein, increasing its negative charge, which may improve the casein micelle protection against the ethanol effects and increase its ethanol stability.

Horne and Parker [49] reported that the removal of calcium ions by EDTA treatment had a reverse effect, shifting the entire profile to lower pH values. These movements of the profile, to higher pH levels when the soluble calcium content was increased and to lower pH when it was reduced by EDTA treatment, were also confirmed in previous studies [50,51,52]. Cases et al. [53] reported that EDTA-treated milk released 33% of the total casein micelles proteins, and the proportion of β-casein in the aqueous fraction was 13% higher than those of the other caseins. Thus, ethanol stability increased for EDTA-treated camel milk, suggesting that ethanol stability is closely dependent on the level of soluble calcium and would be decreased by increasing the free Ca^2+^ concentration [18,54].

In the current study, EDTA samples of pasteurized (heat-treated) camel milk (Figure 2A,B) have better stability than the control. This is because the content of calcium was reduced due to the heat-treatment process. Pingle and Pawar [55] reported that raw goat milk has the highest content of calcium, whereas pasteurized packaged milk has the least. The change in ionic and soluble calcium in the milk sample is linked to the decrease in calcium content after heat treatment; hence, the higher the calcium content cause, the lower the ethanol stability. Furthermore, pasteurized camel milk samples in the current study have better ethanol stability than raw samples (Figure 2). This is might be due to the higher protein content in pasteurized camel milk compared to raw samples (Table 1). These results are in line with Lin et al. [2]. On the other hand, the EDTA-pH profile of raw camel milk samples (Figure 2D,E) was different to that of pasteurized camel milk samples. This could be due to the significant differences in calcium content between pasteurized and raw samples as shown in Table 1. Ye and Harte [15] reported that the behavior of casein micelles, above the concentration of 50% ethanol, was pH, calcium and casein concentration dependent. On the other hand, the EDTA samples of Majaheim (Figure 2C) have better stability than the control at pH below 6.5, whereas, after this pH point, the EDTA and control profiles were similar. This observation cannot be explained on the basis of analysis performed in the current study, which is pending further future investigation.

White and Davies [50] reported that the ethanol stability of milk at its natural pH is closely correlated with the free calcium ion content in the milk. Indeed, the ionic calcium level is the dominant parameter in fixing the position of the ethanol stability profile, regardless of pH value. The same trends were reported for Irish creamery milk [51]. De la Vara et al. [46] found that the ethanol stability of sheep, goat and cow milk was significantly influenced by pH; increased pH from 5.7 to 7.1 increased stability. Meanwhile, Zadow et al. [51] found that the poor ethanol stability of goat milk could be resolved by adjusting the milk’s pH to higher than 7.0 and lowering the ionic Ca^2+^ content by adding EDTA. This could explain the similarity between EDTA-treated milk and control profile samples at a pH level above 6.8; this trend was previously reported by Horne and Muir [56].

Ye and Harte [15] observed that casein micelles aggregated at their isoelectric point and a low ethanol content (<10%), regardless of other factors, such as heating temperature, protein concentration, and even calcium concentration. Indeed, at pH 4.6, the reduction in the electronegative charge of micelles causes the collapse of the κ-casein layer, leading to aggregation/coalescence of the destabilized micelles and the creation of sub-micron-sized aggregates [15,57]. On the other hand, larger aggregates are created at a higher ethanol concentration (20–40%) and at a pH range of 5.0–6.0.

Zhao et al. [18] reported that increasing the pH of Bactrian camel milk would reduce the free calcium concentration in the milk. Hence, ethanol stability increases with increasing pH levels. On the other hand, decreasing milk’s pH enhances the free calcium level, leading to decreased ethanol stability in camel milk [18].

Horne and Parker [49] confirmed that adding NaCl induced a significant reduction in the ethanol stability of milk, resulting from an increase in the non-sedimentable calcium content. Indeed, Horne and Parker [49] reported that NaCl induced a significant reduction in the casein micelles’ electronegative charge, leading to ethanol-induced milk coagulation.

Similar to camel milk, previous studies found that the addition of 2% (*w*/*v*) NaCl to goat milk increased ethanol stability from 40% to 60%, and it further improved when the NaCl concentration increased [23]. These authors explained this behavior by the contribution of the Na/K ratio since the Na/K ratio of goats’ milk is lower than that of bovine milk. The apparent relationship between ethanol stability and the Na/K ratio of sheep, deer, cow, and Bacterian camel milk has been discussed by Zhao et al. [18], Gallego et al. [58], and Park et al. [59] and confirmed by Guo et al. [23]. Although the Na/K ratio was not studied in this research, based on previous observations, the high ethanol stability of camel milk with added NaCl may be due to the high ratio of Na/K.

## 5. Conclusions

This study demonstrated that ethanol stability in camel milk was dependent on the camel breed, pH level, ionic strength, and EDTA addition. Under natural conditions, camel milk samples precipitated upon the addition of an equal volume of ethanol between 50% and 64% ethanol, depending on the camel sample. The ethanol stability of camel milk improved after the addition of NaCl, with the highest values for Majaheim and Alwatania milk, especially at high NaCl concentrations (>5%). Furthermore, camel milk ethanol stability increased with increasing pH (between 5.9 and 7.1) with a sigmoidal trend. The removal of calcium ions by EDTA treatment had a reverse effect, shifting the entire profile to higher ethanol stability values. In future, authors need to use a different concentration of EDTA to understand the EDTA-concentration-pH profile.

## Figures and Tables

**Table 1:** Chemical composition of camel milk from five samples—Alwatania, Alquasim, Majaheim, Wadah, and Hamra (%: g per 100 g of milk sample). ^a–d^ Different letters in the same line indicate significant differences (*p* < 0.05) between camel milk samples.

	Alwatania	(Darir Alabaker) Alquasim	Majaheim	Wadah	Hamra
Fat (%)	3.07 ^a^ ± 0.06	3.07 ^a^ ± 0.06	1.83 ^b^ ± 0.12	2.58 ^c^ ± 0.06	2.40 ^d^ ± 0.00
Protein (%)	3.23 ^a^ ± 0.06	3.00 ^b^ ± 0.10	2.87 ^b^ ± 0.06	2.47 ^c^ ± 0.06	2.43 ^c^ ± 0.06
Lactose (%)	4.87 ^a^ ± 0.06	4.53 ^b^ ± 0.06	4.37 ^c^ ± 0.06	4.40 ^bc^ ± 0.10	4.57 ^b^ ± 0.06
Ash (%)	0.78 ^a^ ± 0.01	0.78 ^a^ ± 0.01	0.78 ^a^ ± 0.01	0.79 ^ab^ ± 0.01	0.80 ^b^ ± 0.01
Total solid (%)	12.10 ^a^ ± 1.0	11.37 ^b^ ± 0.06	10.27 ^c^ ± 0.06	10.17 ^c^ ± 0.06	10.57 ^c^ ± 0.06
Calcium/Cations (ppm)	112.75 ^b^ ± 4.60	111.70 ^b^ ± 1.98	106.95 ^b^ ± 2.62	124.75 ^a^ ± 4.45	128.30 ^a^ ± 4.53
Sodium/Cations (ppm)	217.63 ^bd^ ± 11.77	206.06 ^d^ ± 0	232.78 ^b^ ± 0	261.18 ^a^ ± 5.16	188.64 ^c^ ± 2.19

Note: Data presented as mean ± standard deviation.

## Data Availability

The data presented in this study are available on request from the corresponding author. The data are not publicly available due to privacy.
